# Down syndrome and congenital heart disease: why the regional difference as observed in the Libyan experience?

**DOI:** 10.5830/CVJA-2010-072

**Published:** 2011-11

**Authors:** Z Elmagrpy, A Rayani, A Shah, E Habas, EH Aburawi

**Affiliations:** The Children’s Hospital, Faculty of Medicine, Alfateh Medical University, Tripoli, Libya; The Children’s Hospital, Faculty of Medicine, Alfateh Medical University, Tripoli, Libya; The Children’s Hospital, Faculty of Medicine, Alfateh Medical University, Tripoli, Libya; Medical Department, Faculty of Medicine, Alfateh Medical University, Tripoli, Libya; Department of Paediatrics, Faculty of Medicine and Health Science, United Arab Emirates University, Al-Ain, United Arab Emirates

**Keywords:** congenital heart disease, Down syndrome, geographical difference, Libya

## Abstract

**Background:**

Children with Down syndrome (DS) have about a 40 to 50% incidence of congenital heart disease (CHD). The objectives of this study were to evaluate the distribution and frequency of CHD patterns in Libyan children with DS.

**Methods:**

All patients with DS who were referred to the cardiology clinic between January 1995 and December 2008 were reviewed.

**Results:**

Of the 1 193 patients reviewed, 537 (45%) had an associated CHD. Overall there were 349 (65%) patients who had a single cardiac lesion, and 188 (35%) had multiple cardiac lesions. The most common isolated cardiac lesion was atrial septal defect (ASD), found in 125 (23%) patients, followed by atrioventricular septal defect (AVSD) in 103 (19%), and ventricular septal defect (VSD) in 76 (14%).

**Conclusion:**

Atrial septal defect was the most common cardiac lesion. The distribution of CHDs in Libyan children with DS was similar to what has been reported internationally, but the frequency was not compared with international rates.

## Abstract

Down syndrome (DS) or trisomy 21 is a chromosomal disorder frequently associated with a varied combination of morphological and structural birth defects. These defects are in the form of congenital mental disability, hypotonia, characteristic body features, heart defects, and other systemic congenital malformations. The frequency and severity of these morphological and functional defects vary significantly among affected individuals. The incidence of moderate to severe forms of congenital heart disease (CHD) in Libya is about 5/1 000 live births.^1^

In 1866, John Langdon Haydon Down first characterised DS as a distinct disease with intellectual impairment.^2^ In the late fifties, Lejeune and Jacobs independently reported that DS resulted from an extra chromosome 21. Since then the condition has been known as trisomy 21.^3^,^4^ This particular trisomy is the most common form of chromosomal abnormality, affecting about one in 700 live births.^5^,^6^ It is characterised by the whole chromosomal aneuploidy in about 95% of cases. The remaining 5% is in the form of translocations and mosaics.^7^ The risk of pregnancy with DS increases with the mother’s age, and it can occur with a frequency as high as one in 30 in those older than 45 years.^8^

Approximately 40 to 60% of children with DS have heart defects and among those with CHD, four to 10% are associated with DS.^9^ The cardiac defects are commonly single, but they can be multiple as well. It is highly recommended that DS patients be referred early for cardiac screening. Chest infection, CHD, blood disorders, leukaemia and lymphoma are not uncommon causes of death.^10^,^11^ Nowadays, almost all CHD in DS patients are surgically manageable, with good results. Moreover, the postoperative morbidity and mortality associated with cardiac surgery has fallen dramatically in recent years due to advances in intensive care units, peri-operative care and improved treatment related to respiratory illnesses.^12^,^13^

The aim of this study was to determine the mode of presentation, and type and distribution of CHD affecting DS patients in Libya, and to compare this with previously reported studies.

## Methods

In a retrospective descriptive study, medical records of clinically diagnosed DS patients were reviewed. There were 1 193 children with DS who had presented to Alfateh University Children’s Hospital, Tripoli, between January 1995 and December 2008. After detailed histories and thorough physical examinations were recorded, two-dimensional echocardiography and Doppler studies were performed using the ALOKA SSD-800 before 2005, and the Philips EnVisor C HD, Philips Medical Systems after 2005.

The data were analysed by descriptive statistics, using Microsoft Excel and Minitab version 16 statistical software packages. Previously reported literature was reviewed to compare our results with the international geographical distribution of cardiac defects in patients with DS.

## Results

There were 1 193 patients with DS who attended the cardiac clinic and of these, 537 (45%) had CHD. The male-to-female ratio was 1:1.4. The reasons for presentation are given in Table 1, with the commonest being routine screening (44%). Cardiac murmur was the reason for referral in 177 (33%) patients, heart failure in 62 (12%), chest infection in 36 (7%), and cyanosis in 19 patients (4%).

**Table 1:** Cardiac Evaluations Of The Patients Studied

Number	537
M:F ratio	1:1.4
Reason for cardiac evaluation	
Screening	237 (44%)
Murmur	177 (33%)
Heart failure	62 (12%)
Cyanosis	19 (4%)
Chest infection	36 (7%)
Others	6 (1%)
Age at diagnosing CHD	
0–1 months	149 (28%)
> 1–6 months	230 (43%)
> 6 months – 1 year	66 (12%)
> 1–3 years	44 (8%)
> 3–5 years	18 (3%)
> 5–10 years	19 (4%)
> 10–18 years	11 (2%)

CHD, congenital heart disease.

Of the overall number of patients with CHD, 349 (65%) had a single cardiac lesion, whereas the remaining 188 (35%) had multiple defects. The most common single defect was atrial septal defect (ASD), which was found in 125 (24%) patients, followed by atrioventricular septal defect (AVSD) in 103 (19%), and ventricular septal defect (VSD) in 76 (14%) cases. Patent ductus arteriosus (PDA) occurred in 28 (5%) cases.

Other common defects such as combined ASD plus VSD with other complex lesions was found in 77 (14%) patients, VSD plus PDA was found in 42 (8%), and 40 (7%) had ASD plus PDA. PDA was the most common cardiac defect associated with other cardiac lesions. It was found in association with VSD and ASD in 82 patients (15%) (Table 2).

**Table 2:** The Frequency And Types Of Congenital Heart Disease

*Cardiac lesions*	*Patients, n = 537 (%)*	Age*	*M:F ratio*
Single lesion	349 (65%)	4 months (3 days –18 years)	1:1.4
ASD	125 (23%)	3 months (3 days –15 years)	1.1:1
AVSD	103 (19%)	3 months (3 days – 12 years)	1:1.6
VSD	76 (14%)	4 months (4 days – 14 years)	1:1
PDA	28 (5%)	5 months (1 moth – 18 years)	1:2
TOF	13 (2%)	3 months (3 weeks – 9 months)	1:1
COA	4 (1%)	5 months (1 week – 6 months)	1:0.3
Multiple lesions	188 (35%)	4 months (2 days – 18 years)	1:1.4
ASD with VSD plus others	77 (14%)	1 year (2 days – 18 years)	1:1.5
VSD with PDA plus others	42 (8%)	1.5 months (1–3 months)	1:0.8
ASD plus PDA with others	40 (7%)	4 months (1.5 months – 1.5 years)	1:0.8
ASD with pulmonary stenosis	11 (2%)	30 days (4 days – 3 years)	1:3
Other rare associations	19 (4%)	2 months (2 days – 2 years)	1:1

ASD, atrial septal defect; AVSD, atrioventricular septal defect; VSD, ventricular septal defect; PDA, patent ductus arteriosus; TOF, tetralogy of Fallot; CoA, coarctation of aorta. *Median (range) age at diagnosing congenital heart disease.

The median age (range) at diagnosis with ASD was three months (three days – 15 years) and for those with complex and mixed cardiac lesions it was four months (two days – 18 years) (Table 2). The rare associations of complex CHD were those of double-outlet right ventricle and transposition of the great arteries in two patients, pulmonary atresia with VSD in one patient and a combination of complete atrioventricular septal defect with tetrology of Fallot in three patients.

## Discussion

Approximately 50% of the patients evaluated for DS had an associated CHD. This finding is similar to internationally reported figures.^9^ ASD of secondum type was the most common single congenital cardiac defect and was found in 125 of the 537 DS patients (24%). This finding was not consistent with what has previously been reported in Europe, Sudan and the USA, where ASD was reported to occur in only 5% of the DS patients in Europe and Sudan and 8% in the USA patients.^14^-^16^ ASD of secondum type has been reported in Mexico in 38% and Saudi Arabia in 21% of patients with DS.^17^,^18^ These high distributions are similar to that in our report.

In most of the European countries, Sudan, Turkey and the USA, AVSD was the most common cardiac defect.^14^-^16^,^19^ In Asia on the other hand, VSD was the most common single cardiac defect, followed by ASD or AVSD.^18^,^20^,^21^ In our series, AVSD with a common atrioventricular valve was the second most common CHD, found in 103 (19%) patients. In Guatemala, PDA (29%) followed by VSD (28%) were the most common cardiac defects.^22^

The isolated cardiac lesions represented about 65% of all CHD in our study, compared with 80% in Guatemala,^22^ 74% in Mexico,^17^ and 78% in Turkey^19^ (Table 3). Furthermore, patients’ age at diagnosis with ASD was younger than those with complex and other associated cardiac lesions. This minimised the possible bias that patients with more complex lesions died earlier before diagnosis, compared with those with ASD.

**Table 3:** Comparison Of Congenital Heart Diseases In Libyan Trisomy 21 Patients (*n* = 537) With Other Nations

*Cardiopathy*	*ASD %*	*AVSD %*	*VSD %*	*PDA %*	*TOF %*	*CoA %*	*Others %*
Libya	24	19	12	4	2	1	39
Mexico^17^	38	9	30	21	1	0	1
Guatemala^22^	13	10	28	29	0	0	22
Saudi Arabia^18^	21	23	33	14	5	0	4
India^21^	12	3	26	5	16	0	39
China^20^	13	15	44	12	13	0	2
Italy^14^	5	55	25	5	6	0	4
USA^16^	8	45	35	7	4	1	1
Sudan^15^	5	48	23	7	6	0	11

ASD, atrial septal defect; AVSD, atrioventricular septal defect; VSD, ventricular septal defect; PDA, patent ductus arteriosus; TOF, tetralogy of Fallot; CoA, coarctation of aorta.

On reviewing the literature (Table 3), it appears that the frequency and distribution of CHD in DS varies in different geographical regions. The reason for this difference in the distribution of CHD among DS patients is not clear, although some consistency is observed between certain global areas. This variation in geographical distribution may be caused by numerous factors, one of which could be the genetic make up of each nation or global region; or it could be due to specific embryological mechanisms.

Types of CHD can also be determined by cell characteristics in each nation or population.^23^ The embryology and anatomy of VSD, ASD and PDA are quite different from that of AVSD.^24^-^26^ Some publications have suggested that ethnicity and different geographic factors, such as high altitude with lower partial pressures of oxygen may contribute to a higher frequency of PDA.^17^,^27^,^28^ However, these hypotheses need to be tested by further large-scale multinational collaborative studies.

The most common mode of presentation of CHD in DS was the routine referral to cardiology clinics, but only 44% of the cases were presented in this manner. There were 62 patients (12%) who presented with heart failure; this was probably due to the fact that these patients were missed or not referred at an earlier age for cardiac screening. This stresses the importance of early referral to cardiac clinics.^1^,^29^

The mother’s age is an important risk factor for DS, with the risk of giving birth to a child with DS increasing from 1 in 1 250 at age 25, to 1 in 1 000 at age 30, to 1 in 400 at age 35, to 1 in 100 at age 40, to 1 in 30 at age over 45 years.^30^ Nevertheless, about 80% of infants with DS are born to mothers who are under 35 years of age.^28^ In our series, CHD was more common in children whose mothers were in the age range of 31 to 45 years (71%), which is comparable with international figures. In addition, the single and multiple cardiac lesions were nearly doubled in those of older mothers.

On the other hand, there was no specific association between ASD and mothers’ ages. Interestingly, we found tetrology of Fallot in 13 children of mothers who were over 30 years of age, and not in younger mothers. Coarctation of the aorta is unusual in patients with DS; however, we found this to occur in 1% of the cases, which is similar to a previous report from the USA.^15^

There are a number of limitations in our study, such as the fact that the figures reported herein are not population based, and any patients with DS who died at home or at other hospitals and had never been diagnosed with CHD were not included. Moreover, cytogenetic studies were not routinely performed on all patients because diagnosis was mainly based on clinical grounds. Due to poor organisation and infrastructure at our cardiac centre in previous years, we were unable to accompany the presented figures with any surgical results. Irrespective of these limitations, we believe that we have made some progress in documenting the distribution of CHD in Libyan children with DS.

## Conclusion

This is the first study to document the types, distribution and frequency of CHDs in Libyan children with DS. ASD was the most common single cardiac lesion in DS. The distribution of CHDs in Libyan children with DS was similar to what has been reported internationally but the frequency was not compared with international rates. We stress the importance of early referral and screening for CHDs in this group of patients.
